# Evaluation of the KOWA SL‐19 Plus Portable Slit Lamp With Integrated Digital Camera for Veterinary Ophthalmic Imaging: A Comparative Study With Smartphone‐Based Systems

**DOI:** 10.1111/vop.70018

**Published:** 2025-04-13

**Authors:** Bertrand Michaud

**Affiliations:** ^1^ Clinique Vétérinaire Anima‐Vet Saint‐Genis‐Pouilly France

**Keywords:** imaging, KOWA SL‐19 plus, photography, slit‐lamp, smartphone

## Abstract

**Objective:**

This study compares the performance of the KOWA SL‐19 Plus portable slit lamp, equipped with an integrated digital camera, and a smartphone‐based imaging system adapted to fit on the slit lamp eyepiece for veterinary ophthalmic imaging. Both devices were tested for their efficiency, image quality, and diagnostic utility in clinical settings.

**Animals Studied:**

A total of 20 eyes from 13 animals (7 dogs and 6 cats), presenting various ophthalmic conditions, were examined using both devices.

**Procedure(s):**

During the ophthalmic examination, videos were captured using both the KOWA SL‐19 Plus and the smartphone‐based system, then the best picture was extracted. Extraction time was measured. Thirty‐nine board‐certified ophthalmologists evaluated the images together, focusing on image quality and diagnostic value.

**Results:**

The KOWA SL‐19 Plus outperformed the smartphone‐based system in all categories. The mean extraction time for the KOWA SL‐19 Plus was significantly faster (65.47 s) than for the smartphone system (115.50 s, *p* < 0.000001). Image quality was higher for the KOWA SL‐19 Plus (3.80 vs. 2.98 over 5, *p* < 0.000001), and 85.7% of the images were deemed sufficient for diagnostic purposes, compared to 67.9% for the smartphone‐based system (*p* < 0.0001).

**Conclusions:**

The KOWA SL‐19 Plus is a valuable tool for veterinary ophthalmic imaging, offering superior image quality and faster processing times compared to the smartphone‐based system. It presents a promising step forward for both clinical diagnostics and educational purposes.

## 1 Introduction

Invented in 1911 by Allvar Gullstrand, the slit lamp enables a nearly parallel, intensely bright, and focused beam of light that allows visualizing the anterior segment of the eye with accuracy [1]. Over the years, these devices have undergone numerous improvements, contributing to more accurate and comprehensive eye care [2]. The introduction of digital cameras and their integration in human ophthalmology slit lamps from the early 2000s has led to high‐quality imaging, thus enhancing documentation, monitoring, training, and diagnostic abilities [2, 3, 4]. As smartphones have become increasingly popular and equipped with high‐resolution cameras, various adapters can be used to connect them to slit lamps, facilitating access to high‐quality imaging both in human and veterinary ophthalmology [5, 6, 7, 8, 9].

Unlike human ophthalmology, where integrated cameras in slit lamps are widely represented [10, 11, 12], no such device existed for the portable slit lamps used routinely in veterinary ophthalmology until the Kowa SL‐19 Plus (Kowa—Japan) (named as SL19+ in this report) was introduced in 2024. This device allows real‐time slit lamp image streaming, easy eye picturing, and data export, thereby further improving the comprehension of both clients and students [13, 14].

The aim of this study is to perform a comparison between the SL19+ and a universal smartphone‐based imaging system (SBIS) that utilizes a Xiaomi X12T Pro (Xiaomi Corporation, Beijing, China) mounted on the slit lamp's ocular. We expect this study to prove that the SL19+ will provide better quality images and faster extraction times than the SBIS.

## 2 Material and Methods

The study was conducted at Animavet Veterinary Clinic's ophthalmology department, with ethical approval given by the VetagroSup Lyon ethical committee and informed consent obtained from the owners of the animals involved. Twenty eyes of 13 animals (7 dogs and 6 cats) were included in the study. The cohort was divided into 4 healthy eyes and 16 eyes exhibiting various ophthalmic conditions ranging from corneal ulcers to cataracts (Table 1).

**TABLE 1:** Demographics of the studied population.

Species	Breed	Age (month)	Sex	Neutered	Eye pathology
Dog	Malinois	32	Female	Yes	None
Cat	Domestic Shorthair	135	Male	Yes	OD cataract/OS normal
Cat	Sphynx	120	Male	Yes	OD corneal sequestrum
Dog	French Bulldog	51	Female	Yes	OS corneal graft follow up
Cat	Abyssinian	95	Male	Yes	OS corneal graft follow up
Dog	Labrador retriever	72	Male	No	PRA
Dog	Lhasa Apso	61	Female	Yes	OU prolapse of the nictitating membrane gland
Cat	Domestic Shorthair	40	Female	Yes	OU eosinophilic keratitis
Cat	Sphynx	93	Male	Yes	OD NORMAL/OS CONJUNCTIVAL MELANOMA
Dog	English Springer Spaniel	180	Female	Yes	OU Primary glaucoma
Dog	Beagle	122	Male	No	OU Cataract follow up
Dog	Husky	49	Female	Yes	OU Chronic superficial keratitis
Cat	Domestic Shorthair	136	Female	Yes	OU corneal graft follow up

### 2.1 Imaging Systems

**FIGURE 1:**
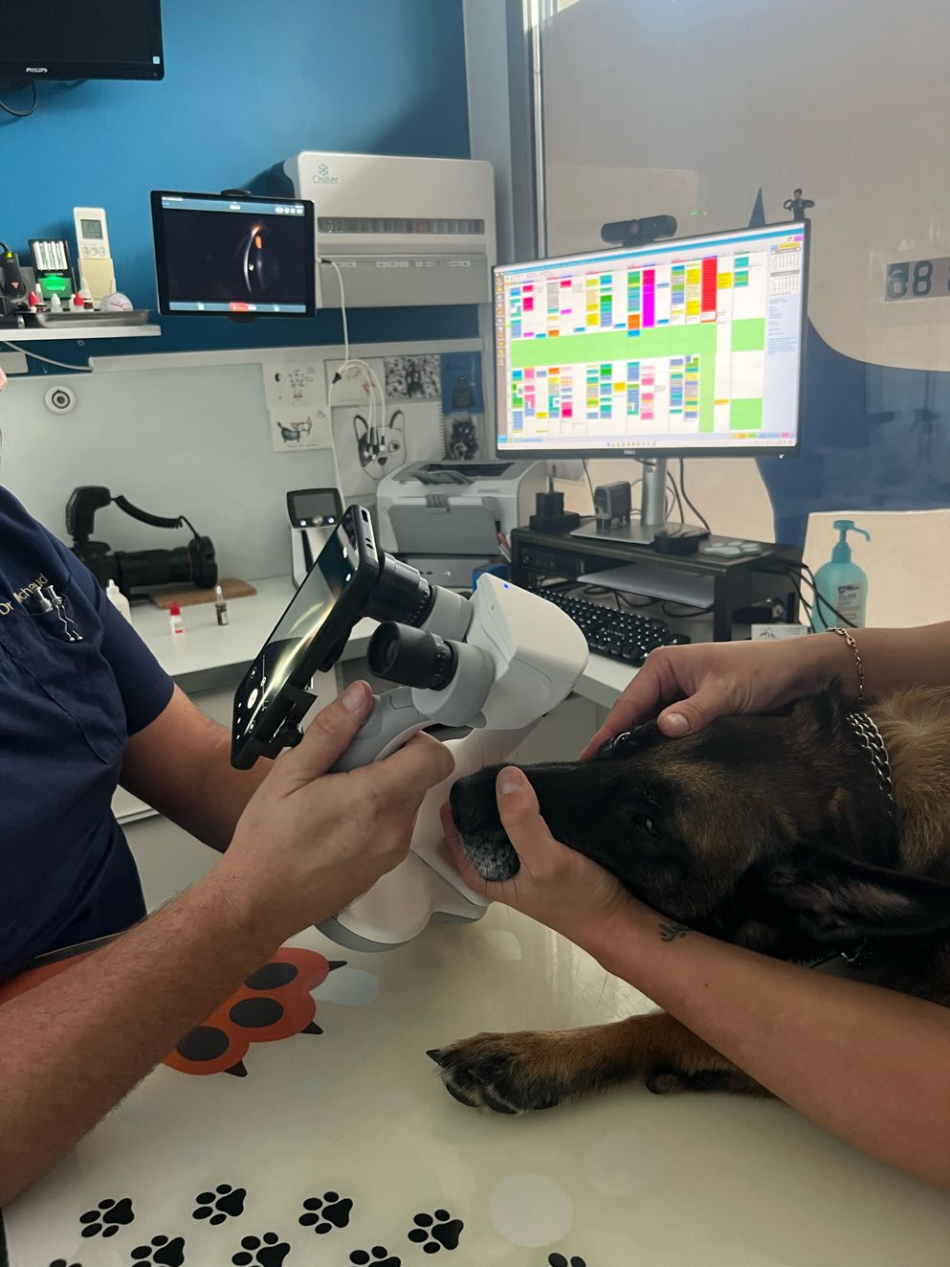
Eye examination with SL19+ with live streaming on an iPad and smartphone recording through the eyepiece of the slit lamp.

The Kowa SL‐19 Plus (Kowa—Japan) is a portable slit lamp equipped with a Full HD 2megapixel CMOS camera, with a maximum speed of 25 frames per second at a resolution of 1920 × 1080 pixels. The biomicroscope is connected via a local Wi‐Fi to an iPad (Apple Inc., Cupertino, CA, United‐States) on which the Kowa‐SL19 application (Kowa—Japan) is installed and broadcasts the livestream of the slit lamp. By pressing the capture button on the slit lamp, the video is recorded on the iPad (Figure 1) and can then be viewed later. The software automatically extracts the best images from the examination performed. The videos and photos can then be transferred to a predefined folder on a computer on the local network with a single click. As the “regular” SL‐19 slit lamp (without camera) background light (3 intensities) can be added, and brightness can be changed; 16× magnification is not available with the SL19+.

#### 2.1.1 The Smartphone Imaging System

A Xiaomi X12T Pro (Xiaomi Corporation, Beijing, China) smartphone with a Leica f/1.7 lens (Leica Microsystems, Wetzlar, Germany) and 200Mp camera was mounted onto one of the eyepieces of the slit lamp with a Smartoscope Vario Universal adaptor (Swarovski Optik—Austria). Videos were recorded in 4 k and at 60 frames per second with the smartphone and then transferred from the smartphone to the computer using WeTransfer (WeTransfer B.V., Amsterdam, Netherlands).

The pictures were then manually extracted by taking a screenshot of the best frame in the video and archived.

### 2.2 Procedure

Both devices were used at the same time when recording the video of the ophthalmic examination performed by the same veterinarian. Images were taken of the adnexas (*n* = 2), cornea (*n* = 13), iris (*n* = 4), irido‐corneal with an ophthalmic Koeppe lens (*n* = 3), lens (*n* = 7) and retina with a Digital Series Wide Field Lens (Volk, Wilmington, Delaware, United States) (*n* = 1). The SL19+ and SBIS pictures were extracted to a personal computer and then renamed and archived by the same operator. Extraction time was measured randomly, using www.randomizer.org to generate a random sequence determining the order in which each device was used first to measure the extraction time and compared for all the pictures; this is the measurement of the time between selecting the media on the device, transferring the file, and renaming the best image on the computer.

### 2.3 Evaluation and Scoring

**FIGURE 2:**
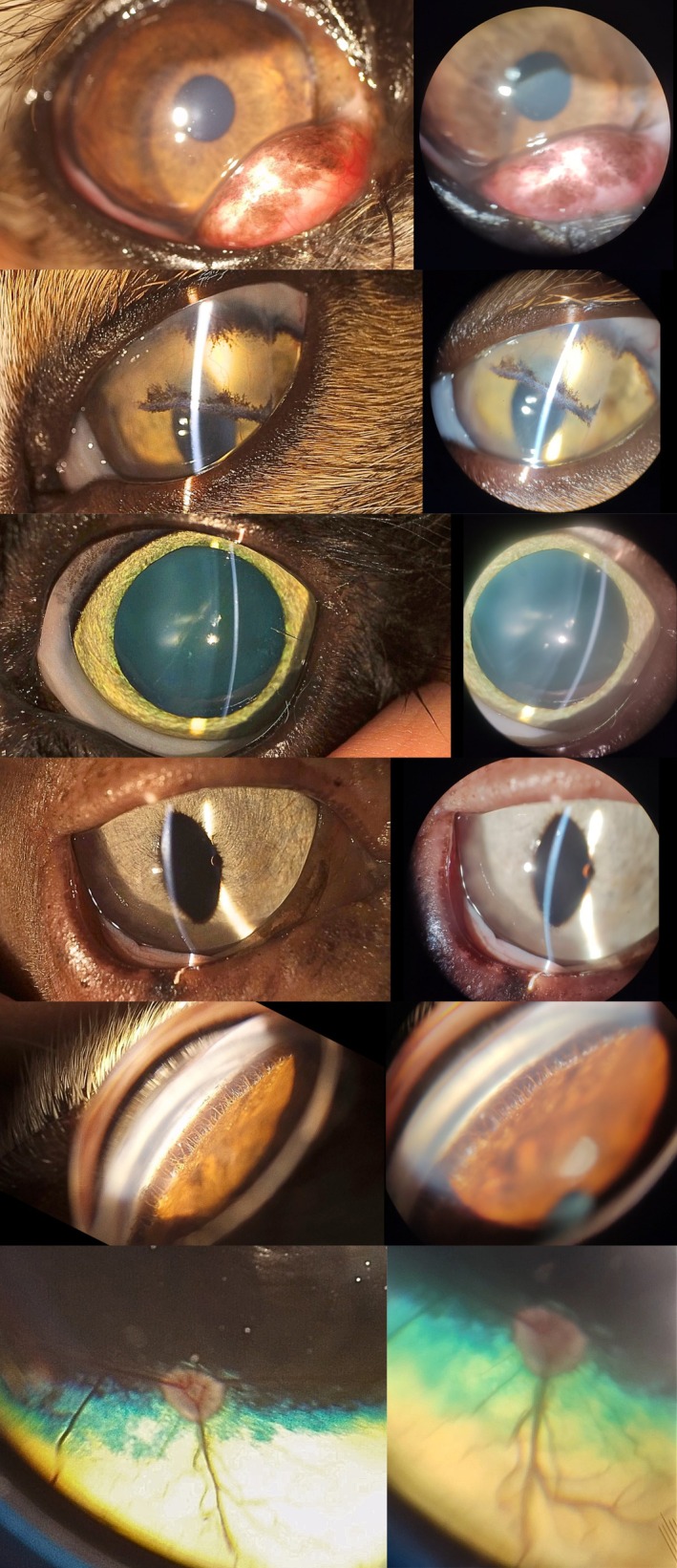
Comparing pictures obtained with the SL19+ (left hand side) and SBIS (right hand side).

We obtained 2 pictures for every examination with both devices (Figure 2). The survey was built using randomly organized pairs of pictures from the same exam with Google Forms (Google LLC, Mountain View, CA, USA) and sent to ECVO and ACVO board‐certified ophthalmologists using Listserv. Respondents were asked to evaluate the 30 cases blinded: for each comparison, they indicated their preferred picture, rated the quality of each image from 1 (ugly) to 5 (very good) and indicated whether the image could allow them to establish a formal diagnostic.

### 2.4 Data Analysis

The data were collected via a Google Forms (Google LLC, Mountain View, CA, USA) survey and included image quality ratings, preferred image choices, and diagnostic confidence. Extraction times were treated separately. Descriptive statistics, including means and standard deviations, were calculated for all variables.

Paired *t*‐tests were used to assess significant differences between the two systems for extraction times, image quality ratings, and diagnostic confidence. The significance level was set at *p* < 0.05. For image preference, the proportion of responses favoring the SL19+ or the smartphone was calculated.

Box plots were generated to visually represent the distribution of photo ratings, diagnostic confidence, and extraction times. Statistical analysis and visualizations were conducted using Python (version 3.13.0), with relevant libraries such as SciPy for statistical testing and Matplotlib for generating the plots.

The paired *t*‐test was chosen for this analysis because the data involved comparing two related conditions by the same group of veterinary ophthalmologists. Since the same respondents provided ratings for both devices, the data were paired, making the paired *t*‐test the most appropriate method to assess differences in extraction times, image quality, and diagnostic confidence. Descriptive statistics were also used to summarize the data and provide an overview of trends before conducting the formal statistical comparisons.

## 3 Results

A total of 39 veterinary ophthalmologists with an average of 16.3 years as a panelist participated in the survey.

### 3.1 Extraction Times

**FIGURE 3:**
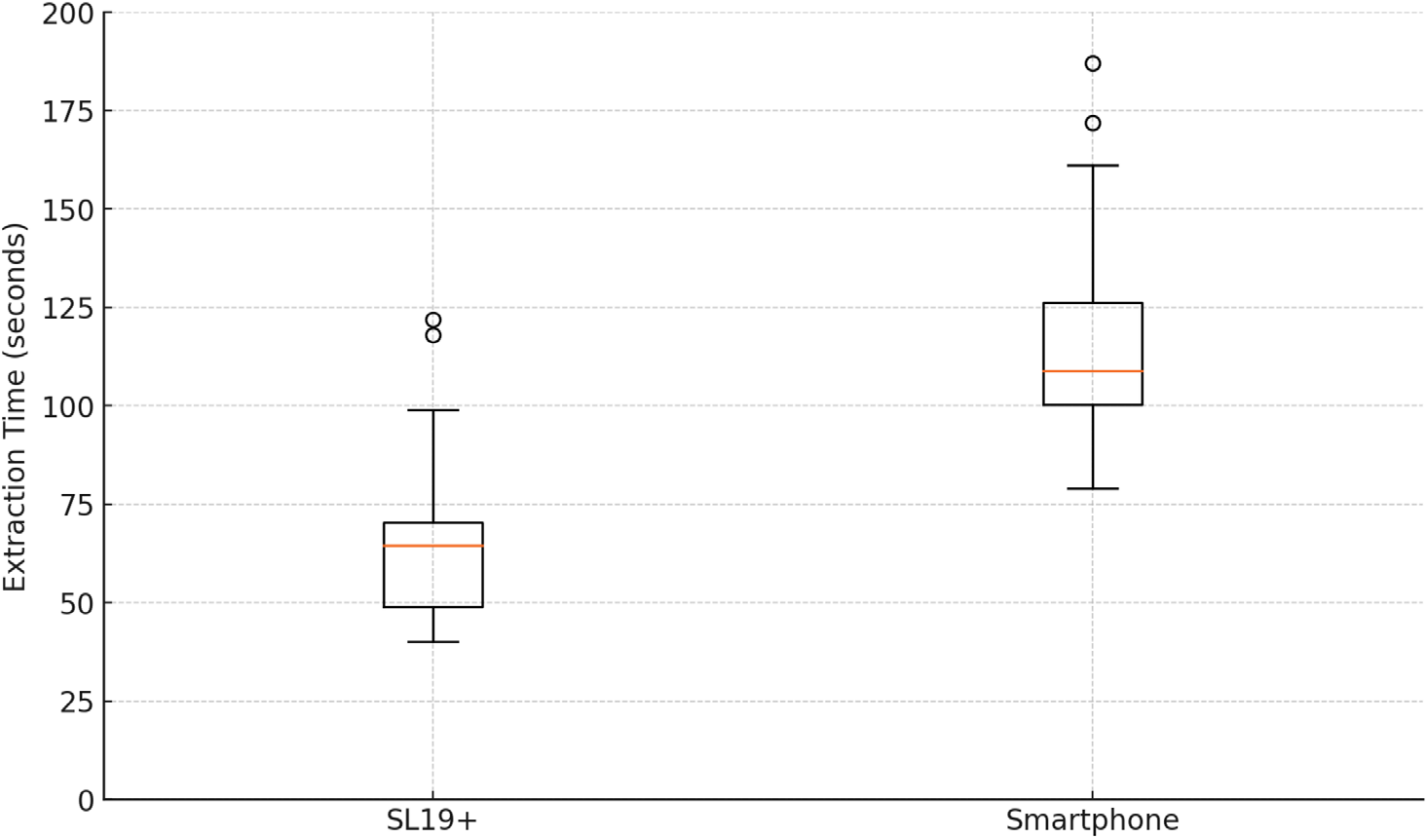
Comparison of extraction times between the SL19+ and smartphone.

The extraction time was significantly faster for the SL19+ compared to the smartphone. The mean extraction time for the SL19+ was 65.47 s (SD = 10.24), while extracting the smartphone pictures took an average of 115.50 s (SD = 13.80); the results are represented in Figure 3. The paired *t*‐test showed a statistically significant difference between the two systems (*p* < 0.000001). This substantial time saving suggests that the SL19+ is much faster in clinical settings.

### 3.2 Picture Preference

Veterinarians overwhelmingly preferred images captured by the SL19+ system. Across the 30 comparisons, images from the SL19+ were selected as superior in 84.0% of cases while images from the smartphone were preferred in only 16.1% of cases. This demonstrates a clear preference for the quality of images provided by the SL19+.

### 3.3 Photo Quality Ratings

**FIGURE 4:**
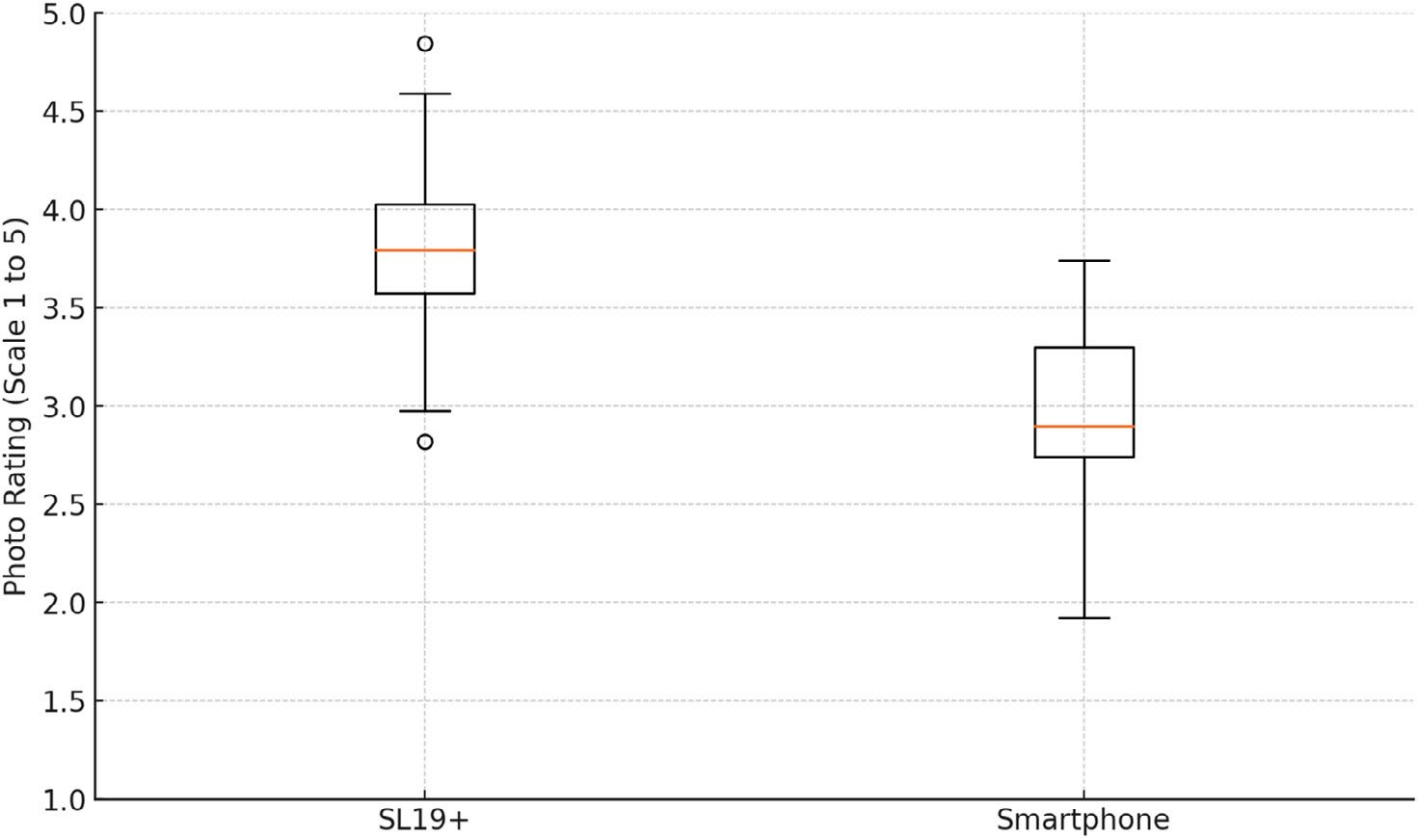
Comparison of photo ratings between the SL19+ and smartphone.

Participants rated the image quality on a scale from 1 to 5. The average rating for the SL19+ images was significantly higher (mean = 3.80, SD = 0.45) compared to the smartphone pictures (mean = 2.98, SD = 0.55). The results are represented as box plots (Figure 4). A paired *t*‐test revealed that this difference was statistically significant (*p* < 0.000001). This indicates that the SL19+ produces higher‐quality images than those obtained using a smartphone.

### 3.4 Diagnostic Confidence

**FIGURE 5:**
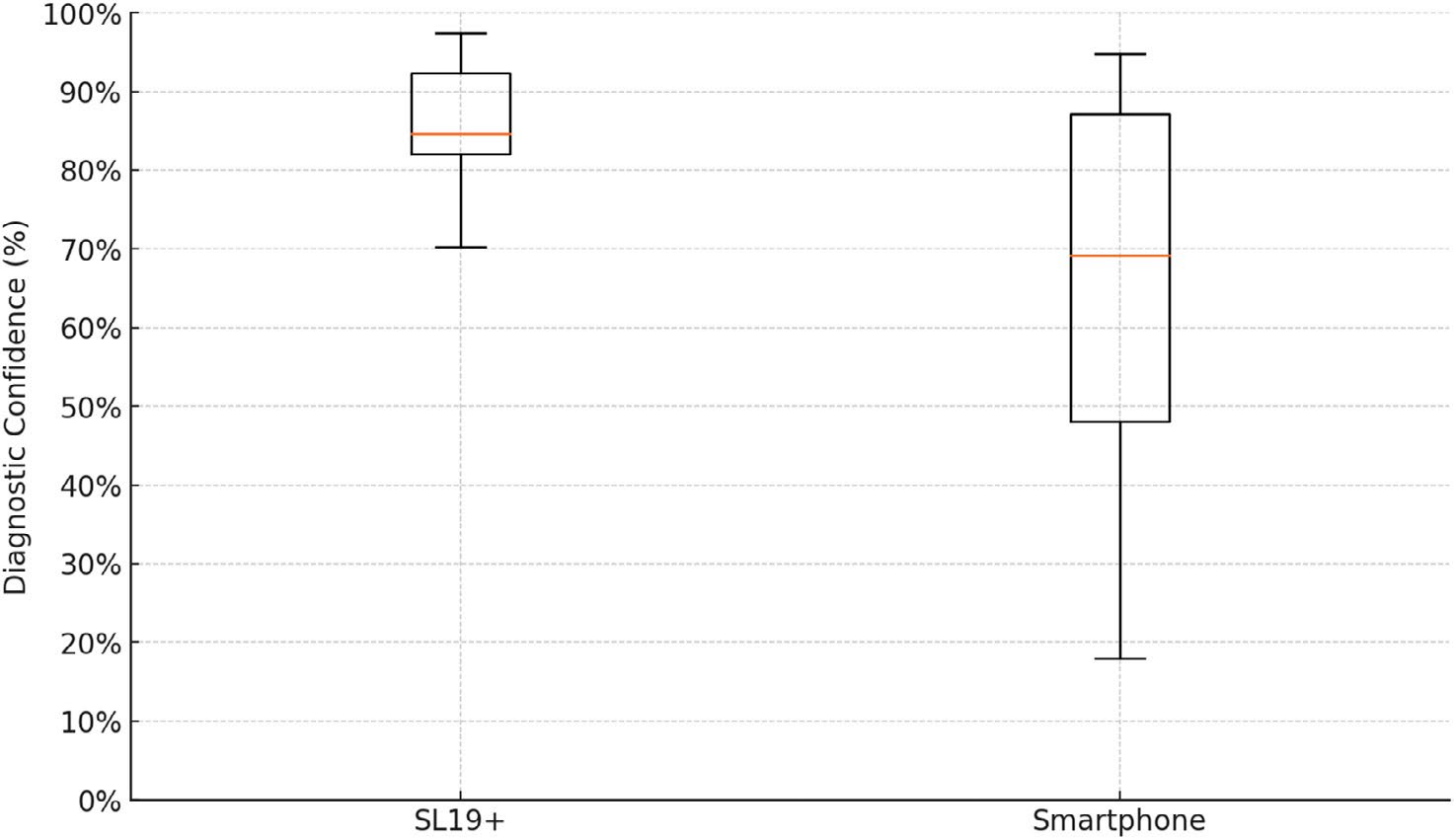
Comparison of diagnostic confidence between the SL19+ and smartphone.

In terms of diagnostic confidence, 85.7% of the SL19+ images were deemed sufficient to make a diagnosis, compared to 67.9% of the smartphone images (Figure 5). The paired *t*‐test showed this difference to be statistically significant (*p* < 0.0001). This suggests that the SL19+ system provides images that are not only preferred but which are also more likely to support accurate clinical diagnoses.

## 4 Discussion

This study highlights the rapid evolution in veterinary ophthalmic imaging technology, comparing two distinct approaches: SL19+ and smartphone‐based slit‐lamp imaging systems. Our findings demonstrate the notable improvements in time efficiency, image quality, and diagnostic confidence when using the SL19+, reflecting broader trends in the progress of slit‐lamp technologies, as reported in previous studies [14, 15]. The ability to quickly acquire, store, and share high‐quality images also enhances the pedagogical value of the SL19+, benefiting veterinary interns, residents, and even pet owners [14, 16].

Our results show that the SL19+ offers faster image extraction compared to the smartphone‐based system used in this study. This aligns with the gains in efficiency described in human ophthalmology [6, 13]. The reduced need to set up, adjust, and handle a separate smartphone attachment further enhances the ease of image acquisition.

Moreover, the SL19+ provides superior image quality. This finding mirrors previous reports of slit‐lamp imaging improvements, where newer digital systems have improved resolution and detail, particularly for anterior segment imaging [4, 12].

However, this study has a major limitation: the relatively small number of examinations (30 images per device), which may not be representative of the full range of diagnostic scenarios encountered in veterinary practice. Expanding the dataset with a larger number of cases, including multiple examples per pathology, would provide a more robust assessment of the two imaging systems and improve the reliability of the findings.

A secondary limitation is the use of one single smartphone for the study; other smartphones may provide better anterior segment images than the Xiaomi. Furthermore, while the Xiaomi X12T Pro Leica‐branded lens is well‐regarded in mobile photography, it cannot be directly compared to dedicated DSLR camera lenses. Image quality is influenced not only by the lens but also by sensor size, noise management, and software processing. This should be considered when interpreting the results.

Finally, the qualitative assessment of image quality by multiple ophthalmologists introduces inherent subjectivity, as individual perception and experience can influence evaluations.

Restricting the evaluation to a smaller number of ophthalmologists could enhance the level of detail and precision in the comparison, allowing for more standardized descriptions and a more refined analysis of the differences between images obtained with each system. This approach could improve consistency while providing a clearer interpretation of the imaging performance of both methods.

The difference in frames per second between the SL19+ (25 fps) and the SBIS (60 fps) may slightly influence the extraction time, as 140% more pictures were generated with the SBIS, resulting in a longer process to isolate the best one. However, the main difference in extraction time is primarily due to the lengthy export process of the massive video files from the smartphone to the computer, as well as the SL19 app, which automatically extracts the best pictures, providing a significant time‐saving advantage.

From our experience, we noted that SL19+ images may sometimes be overexposed, a limitation that has also been observed in other high‐sensitivity ophthalmic cameras [17, 18]. This suggests that further fine‐tuning of image capture settings in veterinary applications may be needed to better represent what our eyes really see through the slit lamp.

The SL19+ images have a much better depth of field and a greater dynamic range compared to the SBIS. This may be improved with other camera phones that have smaller apertures and different lens designs. The gray, washed‐out appearance of the SBIS images may be due to light leakage into the eyepiece of the digiscoping adaptor we used. This issue could be mitigated by using a black foam ring around the camera lens.

Furthermore, the cost of the SL19+ (8500 USD) may be a barrier to widespread use, especially when compared to the lower‐cost universal smartphone adapter (around 200 USD). The SBIS, though cheaper and more portable, frequently encounters constraints regarding compatibility with various phone models and slit lamp types, and these can hinder the standard binocular operation of the slit lamp [8, 9, 15]. Although smartphone‐based systems have been effectively utilized in both human and veterinary medicine [6, 12], they frequently lack the accuracy and user‐friendliness provided by specialized integrated systems [8, 12].

## 5 Conclusion

The SL19+ represents a substantial technological improvement in veterinary ophthalmic imaging, offering improved image quality, faster extraction times, and enhanced utility for both diagnostics and education. However, its high cost may be a barrier to its widespread use. Further studies with a larger, structured dataset and fewer evaluators could refine image comparisons and improve performance assessments. Comparing the SL19+ to other phones and digiscoping systems would also better define its clinical advantages and limitations.

Future developments may allow the integration of a 3D camera [19] to ensure a rendering closer to the reality perceived by the examiner as well as the possibility of adding an indirect ophthalmoscopy lens to allow easy retinal imaging with the slit lamp [16].

## Author Contributions

**Bertrand Michaud:** conceptualization, investigation, funding acquisition, writing – original draft, methodology, validation, visualization, writing – review and editing, software, formal analysis, project administration, data curation, supervision, resources.

## Ethics Statement

This study adhered to the Association for Research in Vision and Ophthalmology Statement for the Use of Animals in Ophthalmic Research and was approved by the Ethics Committee on the Use of Animals of the School of Veterinary Medicine and Agronomy (VetAgroSup). The owners provided informed consent for this study.

## Conflicts of Interest

The author declares no conflicts of interest.

## Data Availability

The data that support the findings of this study are openly available in Figshare at https://www.doi.org/10.6084/m9.figshare.27616920.

## References

[1] S. Schiff‐Wertheimer and M. F. Loisillier , “Notes on the Construction of a Photographic Slit Lamp,” Archives d'Ophtalmologie et Revue Générale d'Ophtalmologie 18 (1958): 833–835. 13628342

[2] M. M. Gellrich , “A New View of the Slit Lamp,” British Journal of Ophthalmology 93 (2009): 272–273. 10.1136/bjo.2008.146803 19174405

[3] V. Vedantham , “Digital Ophthalmic Photography,” Indian Journal of Ophthalmology 52 (2004): 83–84. 15132393

[4] R. Painter , “Slit Lamp Photography: The Basics,” Journal of Visual Communication in Medicine 38, no. 1–2 (2015): 119–123, 10.3109/17453054.2015.1039502. 26203945

[5] J. Chhablani , S. Kaja , and V. A. Shah , “Smartphones in Ophthalmology,” Indian Journal of Ophthalmology 60 (2012): 127–131. 22446908 10.4103/0301-4738.94054 PMC3339072

[6] S. B. Dubbs , K. M. Blosser , and A. C. Richardson , “A Smartphone, a Slit Lamp, and an Ophthalmology Consult,” Clinical Case Reports 7 (2019): 2004–2005, 10.1002/ccr3.2381. 31624627 PMC6787794

[7] C. C. Hester and B. H. Feldmann , “Smart Phoneography—How to Take Slit Lamp Photographs With an iPhone,” 2021, https://eyewiki.org/Smart_Phoneography_‐_How_to_Take_Slit_Lamp_Photographs_with_an_iPhone.

[8] S. Hu , H. Wu , X. Luan , et al., “Portable Handheld Slit‐Lamp Based on a Smartphone Camera for Cataract Screening,” Journal of Ophthalmology 2020 (2020): 1037689, 10.1155/2020/1037689. 32832134 PMC7421715

[9] W. W. Lee , “Slit Lamp Adapters Turn Smartphones Into Clinical Cameras,” 2013, https://www.ophthalmologyweb.com/Featured‐Articles/136817‐Slit‐Lamp‐Adapters‐turn‐Smartphones‐into‐Clinical‐Cameras/.

[10] R. Fogla and S. K. Rao , “Ophthalmic Photography Using a Digital Camera,” Indian Journal of Ophthalmology 51 (2003): 269–272. 14601858

[11] D. A. Fong , “An Introduction to Slit Lamp Photography,” Journal of Ophthalmic Nursing & Technology 3 (1984): 101–108. 6562184

[12] D. R. Muth , F. Blaser , N. Foa , et al., “Smartphone Slit Lamp Imaging—Usability and Quality Assessment,” Diagnostics 13, no. 3 (2023): 423, 10.3390/diagnostics13030423. 36766528 PMC9913954

[13] C. A. Cutolo , C. Bonzano , R. Scotto , et al., “Moving Beyond the Slit‐Lamp Gonioscopy: Challenges and Future Opportunities,” Diagnostics 11 (2021): 2279, 10.3390/diagnostics11122279. 34943516 PMC8700682

[14] M. M. Ford , “A Method for Teaching Depth of Corneal Lesions Using a Portable Slit Lamp (Biomicroscope),” Veterinary Ophthalmology 27 (2024): 286–289, 10.1111/vop.13188. 38409733

[15] J. D. Akkara and K. Anju , “How‐To Guide for Smartphone Slit‐Lamp Imaging,” Kerala Journal of Ophthalmology 31 (2019): 64–71.

[16] M. Arima , T. Majima , S. Tsukamoto , et al., “The Utility of a New Fundus Camera Using a Portable Slit Lamp Combined With a Smartphone,” Acta Ophthalmologica 97, no. 5 (2019): e814–e816. 30767402 10.1111/aos.14049 PMC6767703

[17] L. Sebbag , R. Ofri , D. Arad , K. W. Handel , and O. Pe'er , “Using a Smartphone‐Based Digital Fundus Camera for Screening of Retinal and Optic Nerve Diseases in Veterinary Medicine: A Preliminary Investigation,” Veterinary Record 194, no. 9 (2024): e4088, 10.1002/vetr.4088. 38637964

[18] J. Yuan , H. Jiang , X. Mao , et al., “Slit‐Lamp Photography and Videography With High Magnifications,” Eye & Contact Lens: Science & Clinical Practice 41, no. 6 (2015): 391–397. 10.1097/ICL.0000000000000148 PMC4630077 26020484

[19] O. Solyman , M. Ahmad , K. Arora , A. D. Henderson , and A. Carey , “Stereoscopic Three‐Dimensional (3D) Slit‐Lamp Photography Using a Compact 3D Digital Camera,” Indian Journal of Ophthalmology 69, no. 5 (2021): 1303–1305, 10.4103/ijo.IJO_2037_20. 33913883 PMC8186585

